# Prenatal exposure to bisphenol A alters the transcriptome-interactome profiles of genes associated with Alzheimer’s disease in the offspring hippocampus

**DOI:** 10.1038/s41598-020-65229-0

**Published:** 2020-06-11

**Authors:** Suporn Sukjamnong, Surangrat Thongkorn, Songphon Kanlayaprasit, Thanit Saeliw, Kanlayaphat Hussem, Watis Warayanon, Valerie W. Hu, Tewin Tencomnao, Tewarit Sarachana

**Affiliations:** 1 Age-related Inflammation and Degeneration Research Unit, Department of Clinical Chemistry, Faculty of Allied Health Sciences, Chulalongkorn University, Bangkok, Thailand; 2 SYstems Neuroscience of Autism and PSychiatric disorders (SYNAPS) Research Unit, Department of Clinical Chemistry, Faculty of Allied Health Sciences, Chulalongkorn University, Bangkok, Thailand; 3 Program in Clinical Biochemistry and Molecular Medicine, Department of Clinical Chemistry, Faculty of Allied Health Sciences, Chulalongkorn University, Bangkok, Thailand; 4 Program in Clinical Biochemistry and Molecular Medicine, Department of Clinical Chemistry, Faculty of Allied Health Sciences, Chulalongkorn University, Bangkok, Thailand; 5 Department of Clinical Chemistry, Faculty of Allied Health Sciences, Chulalongkorn University, Bangkok, Thailand; 6 Department of Biochemistry and Molecular Medicine, The George Washington University School of Medicine and Health Sciences, The George Washington University, Washington, DC USA

**Keywords:** Networks and systems biology, RNA, Chemical biology, Developmental biology, Molecular biology, Neuroscience, Systems biology, Environmental sciences, Molecular medicine

## Abstract

Our recent study revealed that prenatal exposure to bisphenol A (BPA) disrupted the transcriptome profiles of genes in the offspring hippocampus. In addition to genes linked to autism, several genes associated with Alzheimer’s disease (AD) were found to be differentially expressed, although the association between BPA-responsive genes and AD-related genes has not been thoroughly investigated. Here, we demonstrated that *in utero* BPA exposure also disrupted the transcriptome profiles of genes associated with neuroinflammation and AD in the hippocampus. The level of NF-κB protein and its AD-related target gene *Bace1* were significantly increased in the offspring hippocampus in a sex-dependent manner. Quantitative RT-PCR analysis also showed an increase in the expression of *Tnf* gene. Moreover, the reanalysis of transcriptome profiling data from several previously published BPA studies consistently showed that BPA-responsive genes were significantly associated with top AD candidate genes. The findings from this study suggest that maternal BPA exposure may increase AD risk in offspring by dysregulating genes associated with AD neuropathology and inflammation and reveal a possible relationship between AD and autism, which are linked to the same environmental factor. Sex-specific effects of prenatal BPA exposure on the susceptibility of AD deserve further investigation.

## Introduction

Alzheimer’s disease (AD) is the most common type of dementia and accounts for approximately 50–75% of dementia cases worldwide^1,2^. AD is caused by progressive brain cell degeneration that affects wide areas of the cerebral cortices and hippocampi of patients, thus leading to various abnormalities, including memory loss, language difficulties, impaired decision making, and lack of other cognitive skills along with behavioral and emotional changes^3,4^. Two major histological findings that are commonly found in the brain of AD patients are the (i) accumulation of extracellular amyloid plaques produced by abnormal aggregation of neurotoxic amyloid-β (Aβ) protein and (ii) intracellular neurofibrillary tangles (NFT) caused by hyperphosphorylation of tau^5,6^. Amyloid plaque formation is often considered an upstream event in the pathogenesis of AD and is derived from the sequential cleavage of amyloid precursor protein (APP) by beta and gamma secretases^7^. The beta-site amyloid precursor protein cleaving enzyme 1 (BACE1), which is the key enzyme responsible for APP proteolysis, initiates the cleavage of APP at the β-secretase site. Only after this cleavage does γ-secretase further process the membrane-anchored C-terminal fragment, thus leading to the production of amyloidogenic 40 or 42 amino acid Aβ peptides^8^. Aβ monomers can assemble into a larger complex to produce intermediate oligomers or protofibrils, which eventually aggregate into Aβ insoluble fibrils and plaques^7^. In addition, a growing number of studies have shown that endogenous factors, such as genetics, aging, high blood pressure, impaired cholesterol metabolism, type 2 diabetes mellitus, and cerebrovascular disease, may cause or increase the risk of AD. In addition, several environmental factors, including endocrine-disrupting compounds (EDCs), cigarette smoke, air pollutants, toxic metals, and pesticides, have also been linked to AD etiology and/or susceptibility^9–14^.

EDCs are exogenous agents that interfere with the endocrine system and disrupt the physiological function of hormones, consequently causing adverse health outcomes. Several studies have presented strong evidence showing that exposure to EDCs can cause human diseases, including diabetes, cancer, fatty liver disease, reproductive disorders, neurodegenerative disorders, and autism spectrum disorder^15–20^. Bisphenol A (BPA) is an EDC that is widely used as an industrial chemical precursor in the manufacture of polycarbonate plastics, epoxy resins, thermal paper, and many other consumer products, such as food and beverage packaging materials^21,22^. The U.S. Food and Drug Administration (FDA) and European Food Safety Authority (EFSA) have determined the No-Observed-Adverse-Effect Level (NOAEL) for BPA to be 5,000 μg/kg BW/day, and Tolerable Daily Intake (TDI) in humans is 50 μg/kg BW/day. The estimated BPA exposure levels from use in food-contacting materials in infants and adults are 2.42 μg/kg BW/day and 0.185 μg/kg BW/day, respectively^23^. Recent studies have shown that exposure to BPA, even at low doses, induced AD-like neurotoxicity^24^, thereby disrupting the synaptic plasticity of the hippocampus^25^ and causing neural degeneration in the substantia nigra^26^. Moreover, BPA can be transferred across the placenta to the fetus^27^ and delivered to offspring through lactation^28^. *In vivo* studies have shown that maternal BPA exposure can disrupt brain functions and induce aggression^29^, anxiety^30^, cognitive deficits^30^, and learning-memory impairment in offspring mice^31^. Recently, our transcriptome profiling analysis of rat hippocampus revealed that maternal BPA exposure dysregulated genes associated with autism in the offspring hippocampus^32^. In addition to genes linked to autism, several genes were also found to be associated with AD. However, the association between BPA-responsive genes in the offspring hippocampus and AD-related genes has not been thoroughly investigated.

Therefore, in this study, we sought to determine the effects of prenatal BPA exposure on the transcriptome-interactome profiles of AD-related genes in the offspring hippocampus using rats as a model. The list of differentially expressed genes in the hippocampus of rat offspring prenatally exposed to BPA were obtained and overlapped with the list of AD candidate genes. A hypergeometric distribution analysis was conducted to determine the association between BPA-responsive genes and genes differentially expressed in AD. Interactome and biological pathway analyses of BPA-responsive genes were reanalyzed to predict interactions, biological functions, and diseases associated with those genes. Moreover, quantitative RT-PCR analysis was performed to further validate the dysregulation of selected AD-related genes in the hippocampus of male and female offspring. In addition, we obtained transcriptome profiles published in the NCBI GEO DataSets database to identify genes that were differentially expressed in response to BPA exposure and determined the association of those genes with AD.

## Results

### Prenatal BPA exposure disrupts transcriptome profiles of genes associated with AD

To examine the effects of prenatal BPA exposure on the expression of genes associated with AD, we obtained transcriptome profiling data from RNA-seq analysis of hippocampal tissues isolated from six independent litters prenatally exposed to BPA (male n = 3; female n = 3) or a vehicle control (male n = 3; female n = 3) from the NCBI Gene Expression Omnibus (GEO) DataSets database (Accession: GSE140298; https://www.ncbi.nlm.nih.gov/gds/). As reported by Thongkorn *et al*. (2019), when all male and female rat pups under the same treatment conditions were combined into one group, as many as 5,624 transcripts corresponding to 4,525 genes were significantly differentially expressed in the hippocampal tissues of BPA-treated rats (Supplementary Table S1). When each sex was analyzed separately, a total of 2,496 transcripts (corresponding to 1,633 genes) and 4,021 transcripts (corresponding to 2,780 genes) in the hippocampal tissues of BPA-treated male and female pups, respectively, were significantly differentially expressed compared to those in the hippocampal tissues of controls (P-value < 0.05 and FDR < 0.05, (Supplementary Tables S2,S3).

To determine whether these lists of DEGs are associated with AD candidate genes, we obtained a list of genes previously reported to be dysregulated in AD patients from AlzBase (http://alz.big.ac.cn/alzBase/). AlzBase is an online database that provides information on the frequency of gene dysregulation in AD brains, which was collected from previously published studies of the AD brain transcriptome. To include only AD candidate genes that are highly reproducible in independent experiments, we obtained a list of 109 genes reported in at least 15 AD studies and identified as “top ranked genes” by AlzBase (Supplementary Table S4). A comparison of the lists of DEGs to the list of AD genes showed that when all male and female rat pups under the same treatment conditions were combined into one group, as many as 26 transcripts corresponding to 24 DEGs were found to be among the top-ranked candidate genes for AD. Moreover, when each sex was analyzed separately, as many as 20 and 31 DEGs were found to be AD candidate genes in male and female pups, respectively. Among the DEGs that overlapped with AD genes, as many as 15, 18, and 12 DEGs from the combined group, males, and females, respectively, were expressed in the same direction of dysregulation as in the AlzBase database (Supplementary Table S5). Next, the associations between BPA-responsive DEGs and AD candidate genes were assessed via a hypergeometric distribution analysis. Interestingly, when each sex was analyzed separately, DEGs from male and female hippocampal tissues exhibited significant enrichment (P-value < 0.05) in AD-related genes from the AlzBase database (Table 1).

**Table 1:** Association analysis between DEGs responsive to BPA in the offspring hippocampus and top-ranked genes associated with Alzheimer’s disease identified by AlzBase.

List of DEGs from Rat Hippocampus	#DEGs	Number of Overlapping Genes with AlzBase	Hypergeometric P-value	List of Overlapping Genes with Alzbase
Both Sexes	4,525	24	0.925	*ACACB, AEBP1, BSCL2, DYNC1I1, FIBP, FSTL1, GABRA1, GFAP, GOT1, GPI, HBB, ITPKB, MAL2, NELL2, NRSN1, NSF, PCP4, PGK1, RCAN2, SCG2, SCG5, SLC14A1, SNX10, TJP2*
Male	1,633	20	**0.011**	*AEBP1, CCK, CRYM, FOXO1, GPRASP1, HBB, INA, ITPKB, KIFAP3, MAL2, MLLT11, NRSN1, NSF, PGK1, RIT2, RPH3A, SASH1, SNAP25, SNX10, TXNIP*
Female	2,780	31	**0.005**	*ABCA1, AEBP1, AMPH, ATP1A1, BCL6, CCK, CDK5, DYNC1I1, EMP1, FOXO1, FSTL1, GFAP, HBB, ITPKB, MAL2, MLLT11, NCALD, NELL2, NME1, NSF, NUPR1, PCP4, SCG2, SNAP25, SNX10, STMN2, SVOP, TGFBR3, TMEM14A, TRIP10, UCHL1*

Hypergeometric distribution analyses were used to analyze associations between significant DEGs responsive to BPA and top-ranked Alzheimer’s candidate genes obtained from AlzBase. Statistically significant associations were determined by hypergeometric distribution analysis. P-value < 0.05 is considered as significant.

### BPA-responsive genes in the offspring hippocampus are involved in biological functions, pathways, and networks associated with AD

The lists of DEGs in the hippocampus of neonates prenatally exposed to BPA based on a RNA-seq analysis of both sexes of rats, only males, or only females were analyzed using Ingenuity Pathway Analysis (IPA) software to predict canonical pathways, diseases/disorders, biological functions, and upstream regulators significantly associated with DEGs. We found that BPA-responsive DEGs in the hippocampus were significantly associated with “neurological disease”, “nervous system development and function”, and “inflammatory response” (Table 2). Canonical pathways known to be involved in AD and neuroinflammation, such as “synaptogenesis signaling pathway” and “NF-κB activation”, were also predicted to be associated with DEGs. Interestingly, upstream regulator prediction revealed that DEGs in the hippocampus are putative targets of several AD-related proteins, including “TGFB1”, “MAPT”, and “APP”, as well as beta estradiol (P-value < 0.05; Table 2). IPA analysis also identified DEGs involved in specific neurological diseases as well as nervous system development and functions that are implicated in AD (Table 3). We found that when all male and female rat pups under the same treatment conditions were combined into one group, DEGs were significantly associated with “cognitive impairment”, “progressive neurological disorder”, and “dementia”. DEGs in the male and female pups were associated with “Alzheimer’s disease” and other AD-related neurological conditions, including “cognitive impairment” and “tauopathy”. In addition, several nervous system functions, including “memory”, “neuritogenesis”, and “synaptic transmission”, were also significantly associated with DEGs in males and females.

**Table 2:** Gene ontology analysis of DEGs in the hippocampus of rats prenatally exposed to BPA.

Categories	P-value	# DEGs
**Both Sexes**		
***Canonical Pathways***		
Calcium Signaling	3.98E-05	63
Protein Kinase A Signaling	3.98E-04	103
AMPK Signaling	1.05E-03	59
NRF2-mediated Oxidative Stress Response	1.51E-03	53
Synaptogenesis Signaling Pathway	3.31E-03	79
PI3K/AKT Signaling	3.47E-03	48
NF-κB Activation	3.98E-03	26
Apoptosis Signaling	4.47E-03	30
***Diseases/Disorders***		
Cancer	9.74E-129 - 9.16E-06	3,984
Endocrine System Disorders	2.58E-113 - 6.62E-06	3,427
Gastrointestinal Disease	1.85E-109 - 6.12E-06	3,601
Immunological Disease	5.56E-16 - 9.16E-06	1,414
Neurological Disease	8.77E-14 - 8.66E-06	1,360
***Biological Functions***		
Lipid Metabolism	5.31E-13 - 8.56E-06	602
Nervous System Development and Function	3.64E-10 - 5.62E-06	744
Connective Tissue Development and Function	4.38E-10 - 4.16E-06	519
Inflammatory Response	2.37E-08 - 3.67E-06	691
Free Radical Scavenging	2.03E-06 - 5.61E-06	188
***Predicted Upstream Regulators***		
Levodopa	3.27E-12	206
TP53	8.85E-10	480
HNF4A	4.04E-09	527
TGFB1	6.09E-09	455
beta-estradiol	2.88E-07	492
**Male**		
***Canonical Pathways***		
Synaptogenesis Signaling Pathway	3.16E-07	47
Glutamate Receptor Signaling	2.29E-05	14
Circadian Rhythm Signaling	1.45E-03	8
IL-15 Production	1.51E-03	18
Axonal Guidance Signaling	1.62E-03	51
Amyotrophic Lateral Sclerosis Signaling	2.51E-03	15
Huntington’s Disease Signaling	3.55E-03	28
GABA Receptor Signaling	5.37E-03	14
***Diseases/Disorders***		
Cancer	6.33E-114 - 8.47E-10	2,468
Endocrine System Disorders	6.33E-114 - 7.38E-10	2,160
Gastrointestinal Disease	2.76E-106 - 6.03E-10	2,273
Reproductive System Disease	3.83E-67 - 7.88E-10	1,737
Neurological Disease	1.89E-31 - 5.17E-10	1,094
***Biological Functions***		
Nervous System Development and Function	2.76E-41 - 7.39E-10	800
Cellular Growth and Proliferation	6.39E-40 - 5.77E-10	772
Cell Death and Survival	8.03E-31 - 4.75E-11	920
Embryonic Development	5.48E-28 - 7.39E-10	713
Behavior	3.18E-20 - 3.16E-11	231
***Predicted Upstream Regulators***		
MAPT	1.28E-11	66
HTT	1.09E-07	89
BDNF	2.79E-07	49
APP	2.01E-06	105
LMNB1	3.23E-06	15
**Female**		
***Canonical Pathways***		
Synaptogenesis Signaling Pathway	1.00E-12	82
Reelin Signaling in Neurons	4.27E-09	40
Calcium Signaling	6.03E-08	52
Axonal Guidance Signaling	9.33E-08	97
AMPK Signaling	9.77E-07	50
Glutamate Receptor Signaling	3.80E-06	20
Amyloid Processing	7.59E-06	18
Synaptic Long Term Potentiation	7.24E-05	31
***Diseases/Disorders***		
Cancer	6.33E-114 - 8.47E-10	2,468
Endocrine System Disorders	6.33E-114 - 7.38E-10	2,160
Gastrointestinal Disease	2.76E-106 - 6.03E-10	2,273
Reproductive System Disease	3.83E-67 - 7.88E-10	1,737
Neurological Disease	1.89E-31 - 5.17E-10	1,094
***Biological Functions***		
Nervous System Development and Function	2.76E-41 - 7.39E-10	800
Cellular Growth and Proliferation	6.39E-40 - 5.77E-10	772
Cell Death and Survival	8.03E-31 - 4.75E-11	920
Embryonic Development	5.48E-28 - 7.39E-10	713
Behavior	3.18E-20 - 3.16E-11	231
***Predicted Upstream Regulators***		
TGFB1	5.31E-23	364
beta-estradiol	1.03E-18	382
TP53	8.38E-15	345
APP	1.58E-14	195
ERBB2	2.85E-13	163

Canonical pathways, diseases/disorders, biological functions, and upstream regulators significantly associated with DEGs in the hippocampus of offspring rats prenatally exposed to BPA were predicted using Ingenuity Pathway Analysis (IPA) software. Results are shown for the separate analyses of the combined group of males and females, males only, and females only. P-values were calculated using Fisher’s exact test. P-value < 0.05 is considered as significant. A range of P-values are shown for the various subcategories of diseases/disorders or biological functions under a general category, such as cancer or lipid metabolism.

**Table 3:** Neurological diseases and functions significantly associated with DEGs in the hippocampus of rats prenatally exposed to BPA.

Categories	P-value	# DEGs
**Both Sexes**		
***Neurological Diseases***		
Autosomal recessive neurological disorder	6.75E-13	267
Cognitive impairment	1.65E-10	270
Motor dysfunction or movement disorder	3.14E-10	429
Progressive neurological disorder	2.45E-08	373
Dementia	5.84E-06	228
***Nervous System Development and Functions***		
Development of neurons	9.84E-09	316
Neuritogenesis	1.71E-07	240
Morphology of cerebellar cortex cells	2.84E-06	32
Synaptic transmission	4.23E-06	130
Morphology of Purkinje cells	4.58E-06	31
**Male**		
***Neurological Diseases***		
Cognitive impairment	1.76E-23	158
Mental retardation	2.10E-17	104
Movement disorders	1.14E-15	204
Alzheimer disease	1.97E-06	94
Tauopathy	5.91E-06	95
***Nervous System Development and Functions***		
Development of neurons	5.42E-27	192
Neuritogenesis	7.83E-25	154
Proliferation of neuronal cells	9.97E-13	111
Synaptic transmission	1.16E-09	34
Memory	3.95E-09	59
**Female**		
***Neurological Diseases***		
Cognitive impairment	3.81E-23	226
Movement disorders	1.44E-22	334
Mental retardation	1.93E-13	137
Tauopathy	1.18E-12	174
Alzheimer disease	3.85E-12	167
***Nervous System Development and Functions***		
Development of neurons	6.39E-40	310
Neuritogenesis	6.61E-37	248
Proliferation of neuronal cells	8.96E-25	197
Synaptic transmission	7.6E-12	108
Memory	2.43E-11	91

The list of DEGs in the hippocampus of offspring rats prenatally exposed to BPA from RNA-seq analysis were analyzed using Ingenuity Pathway Analysis (IPA) software to predict neurological diseases and functions associated with DEGs. Results are shown for the separate analyses of the combined group of males and females, males only, and females only. P-values were calculated using Fisher’s exact test. P-value < 0.05 is considered as significant.

**Figure 1:**
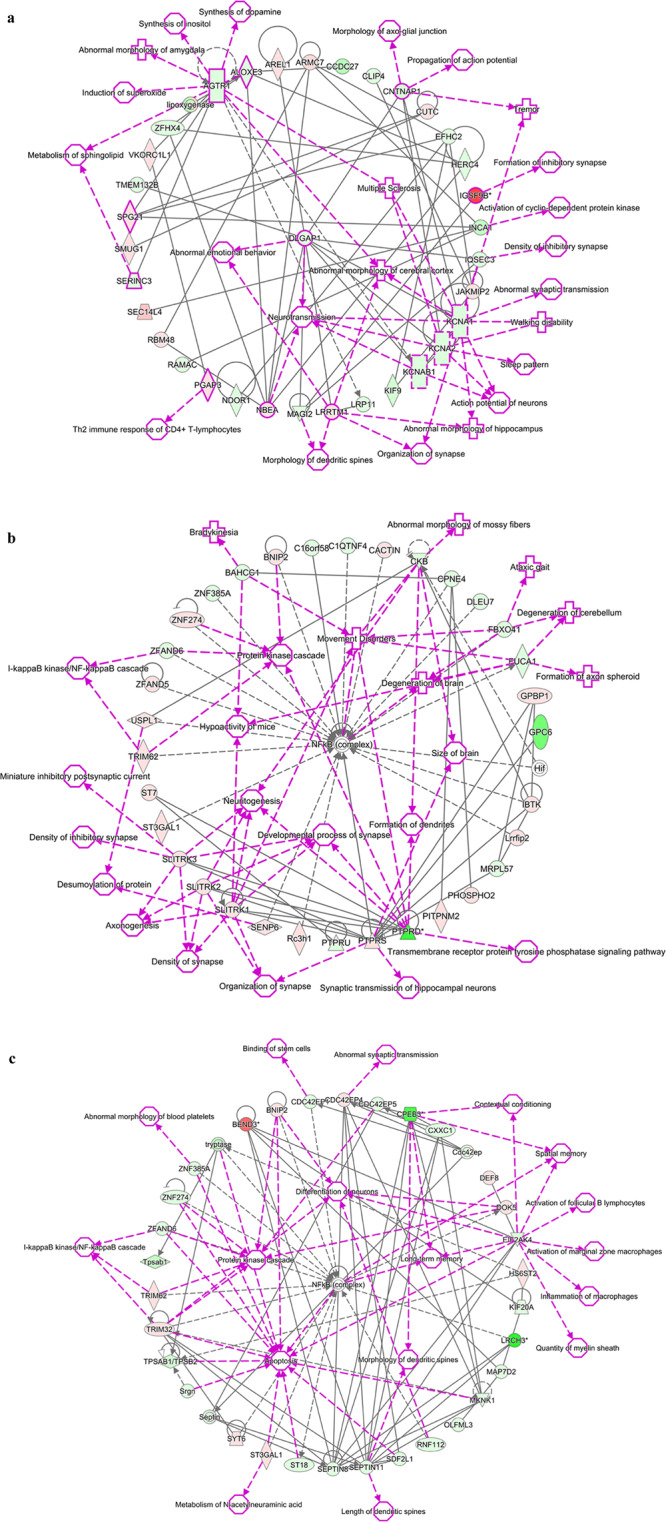
Interactome analysis of genes differentially expressed in the hippocampus of offspring rats prenatally exposed to BPA when using rat offspring consisting of both sexes (**a**), only males (**b**), and only females (**c**), as predicted by Ingenuity Pathway Analysis. Red = up-regulation; Green = down-regulation.

Biological networks of BPA-responsive DEGs were also predicted using IPA software. A representative interactome network of DEGs in the offspring hippocampus showed that when all male and female rat pups under the same treatment conditions were combined into one group, gene interactions among DEGs were associated with several disorders and diseases, including “abnormal synaptic transmission”, “abnormal morphology of hippocampus”, and “abnormal emotional behavior” (Fig. 1a). The interactome network of DEGs in the male hippocampus showed associations with disorders/diseases and neurological functions, including “degeneration of brain”, “movement disorders”, “neuritogenesis”, and “I-kappaB kinase/NF-kappaB cascade” (Fig. 1b), whereas interactome network of DEGs in the female hippocampus showed associations with “abnormal synaptic transmission”, “spatial memory”, “long-term memory”, “inflammation of macrophages” “apoptosis” and “I-kappaB kinase/NF-kappaB cascade” (Fig. 1c). Interestingly, the hub gene in the interactome generated using DEGs from the male and female hippocampus was nuclear factor kappa B (NF-κB), which is the key protein responsible for inflammation and several neurodegenerative diseases, including AD, Parkinson’s, and Huntington’s diseases^33^. These findings suggest that prenatal BPA exposure may alter the expression of genes in the brain through NF-κB protein.

### Prenatal BPA exposure increases NF-κB protein and *Tnf* gene expression in the offspring hippocampus

**Figure 2:**
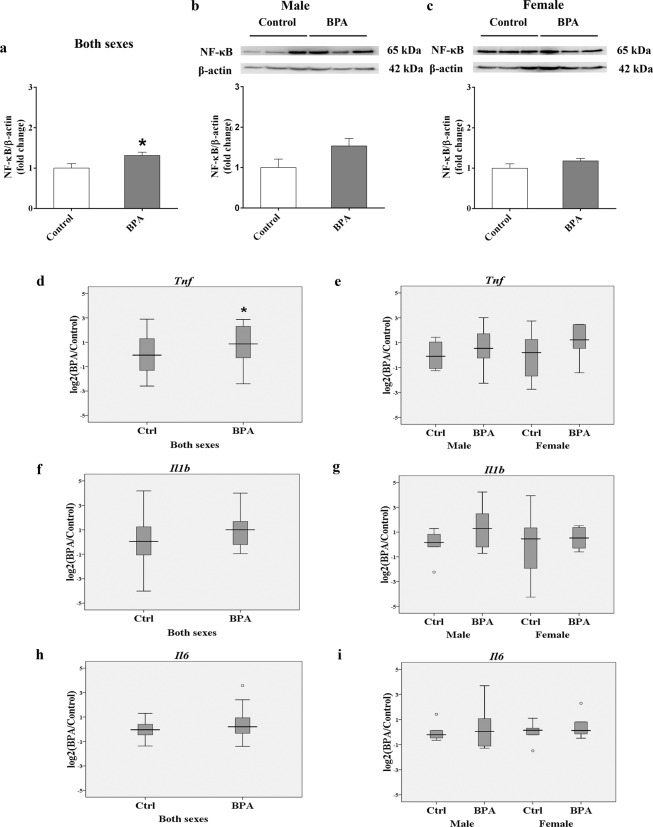
NF-κB and inflammatory cytokines associated with Alzheimer’s disease were increased in the hippocampi of rats prenatally exposed to BPA. The protein level of NF-κB (**a**–**c**) and the expression of *Tnf* (**d**,**e**), *Il1b* (**f**,**g**), and *Il6* (**h**,**i**) were determined in both sexes and separately in the hippocampus of offspring rats prenatally exposed to BPA (male n = 6; female n = 6) or vehicle control (male n = 6; female n = 6). The differences between two groups were analyzed by two-tailed Student’s t-test. P-value < 0.05 is considered as significant.

Because neuroinflammation is highly implicated in the neurodegenerative process of AD^34,35^ and our interactome network analysis revealed that NF-κB protein may play an important role in BPA-mediated neurotoxicity, we further assessed the level of NF-κB protein in the offspring. Interestingly, a Western blot analysis of hippocampal tissues from rats prenatally exposed to BPA (male n = 6; female n = 6) or vehicle control (male n = 6; female n = 6) revealed that NF-κB was significantly increased (P-value < 0.05; Fig. 2a, Supplementary Fig. S1). By analyzing each sex separately, we found that the level of NF-κB exhibited sex differences in response to BPA, being up-regulated in males but not in females (Fig. 2b,c). Moreover, the expression of genes encoding AD-related proinflammatory cytokines, including tumor necrosis factor alpha (TNF-α), interleukin-1beta (IL-1β), and interleukin-6 (IL-6), were also determined (Fig. 2d–i). A quantitative RT-PCR analysis of hippocampal tissues isolated from offspring rats prenatally exposed to BPA (male n = 6; female n = 6) or vehicle control (male n = 6; female n = 6) showed that *Tnf* expression was significantly increased in the BPA treatment group when both sexes were combined. No changes in the expression of *Il-1β* and *Il-6* were observed.

### Prenatal BPA exposure increases the expression of the *Bace1* gene in the offspring hippocampus

**Figure 3:**
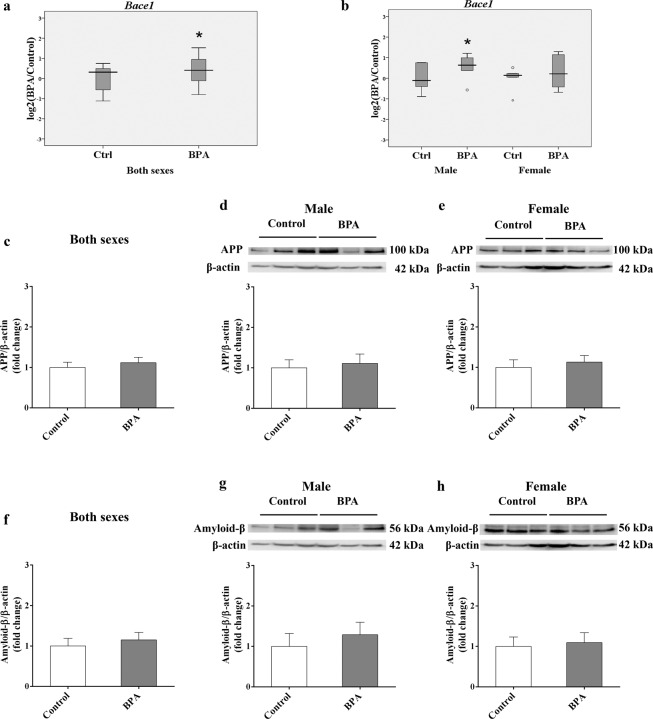
Prenatal BPA exposure increased the expression of *Bace1* in the rat hippocampus. The expression levels of *Bace1* (**a**,**b**) and the protein levels of APP (**c**,**e**) and Aβ (**f**–**h**) were determined in the hippocampus of rats prenatally exposed to BPA (male n = 6; female n = 6) or vehicle control (male n = 6; female n = 6). The differences between two groups were analyzed by two-tailed Student’s t-test. P-value < 0.05 is considered as significant.

Neuronal Aβ accumulation is considered an upstream event in AD pathogenesis, which arises from the improper cleavage of APP, resulting in Aβ monomers that aggregate to form oligomeric Aβ, which eventually aggregate into Aβ fibrils and plaques. In this study, BACE1, a key enzyme that is responsible for APP proteolysis and Aβ generation in AD, was evaluated. We found that *Bace1* expression was significantly increased in the hippocampus of rats prenatally exposed to BPA (P-value < 0.05; Fig. 3a). Interestingly, the up-regulation of *Bace1* was observed in males while no significant changes were found in females when each sex was analyzed separately, indicating that BPA regulates *Bace1* expression in a sex-dependent manner (Fig. 3b). To determine whether increased *Bace1* expression could lead to changes in APP and Aβ protein levels, a Western blot analysis of the offspring hippocampus was performed. Although changes in APP and Aβ protein levels were not statistically significant, it is interesting to note that Aβ tended to increase in the hippocampus of male neonates prenatally exposed to BPA (Fig. 3c–h).

### Integration of data from multiple transcriptomic studies revealed an association between BPA-responsive genes and AD candidate genes

To determine whether BPA-responsive genes identified by other independent investigators were also associated with AD, a total of 12 transcriptome profiling datasets from eight studies using cell lines, primary cells, or tissues from animal models treated with BPA were obtained from the NCBI GEO DataSets database (https://www.ncbi.nlm.nih.gov/gds/). Details of these BPA transcriptome studies were provided in Supplementary Table S6. We then compared the list of DEGs from each study with the list of top-ranked genes associated with Alzheimer’s disease identified by AlzBase. Hypergeometric distribution analyses were used to analyze the associations between BPA-responsive genes from other transcriptome profiling studies and Alzheimer’s candidate genes (Table 4). Interestingly, several AD candidate genes were found to be differentially expressed in response to BPA and the hypergeometric distribution analyses revealed that the lists of BPA-responsive genes identified from as many as five datasets obtained from four BPA transcriptome studies exhibited significant enrichment (P-value < 0.05) in AD-related genes from the AlzBase database, with the highest association in placental tissues (P-value = 4.79E-06).

**Table 4:** Association analysis of BPA-responsive genes identified by other transcriptome profiling studies and top-ranked genes associated with Alzheimer’s disease identified by AlzBase.

GSE DataSets	Sample Types	#DEGs	Number of Overlapping Genes with AlzBase	Hypergeometric Distribution P-value	List of Overlapping Genes with Alzbase	Overlapping Genes with Our DEGs		
						All (both sexes)	Male	Female
GSE44387	Mouse fetal mammary glands (stroma)	1,812	11	**0.004**	*RGS4, INA, ST6GALNAC5, GABRA1, SGIP1, CD44, NMNAT2, ACACB, SLC14A1, CXCR4, AEBP1*	*SLC14A1, ACACB, AEBP1, GABRA1*	*AEBP1, INA*	*AEBP1*
	Mouse fetal mammary glands (epithelium)	2,858	11	0.085	*CALB1, TRIP10, ATP6V1B2, RAB3A, CDK5, STMN2, GRAMD3, TGFBR3, ATP1A1, INA, MRPL15*	*—*	*INA*	*TRIP10, ATP1A1, TGFBR3, CDK5, STMN2*
GSE63852	Mouse placental tissues (high—dose BPA treatment)	1,681	16	**4.79E-06**	*NEFL, CD44, RPH3A, SASH1, MAL2, PGK1, GFAP, OXCT1, MRPL15, RAB3A, MDH1, STMN2, AEBP1, GABRD, NUPR1, BSCL2*	*GFAP, AEBP1, PGK1, BSCL2, MAL2*	*SASH1, AEBP1, RPH3A, PGK1, MAL2*	*STMN2, GFAP, AEBP1, NUPR1, MAL2*
	Mouse placental tissues (low-dose BPA treatment)	982	10	**1.91E-04**	*CHGB, PGK1, GAD1, GFAP, CALB1, UCHL1, BEX4, NCALD, AEBP1, NSF*	*GFAP, PGK1, NSF, AEBP1*	*PGK1, NSF, AEBP1*	*NCALD, GFAP, NSF, AEBP1, UCHL1*
GSE58642	Mouse fetal germ cells (female)	1,340	6	0.229	*KIFAP3, CD200, CD44, MLLT11, SGIP1, KANK2*	*—*	*KIFAP3, MLLT11*	*MLLT11*
	Mouse fetal germ cells (male)	2,303	11	0.095	*SYT1, NFKBIA, NRSN1, PCP4, SST, GRAMD3, FOXO1, GEM, ABCA1, ACACB, SYTL4*	*NRSN1, ACACB, PCP4*	*NRSN1, FOXO1*	*ABCA1, FOXO1, PCP4*
GSE50527	Human osteosarcoma cells (short-term BPA treatment)	3,056	17	**6.20E-04**	*NRSN1, GOT1, ATP6V1G2, RGS7, SASH1, AEBP1, SLC12A7, RGS4, SVOP, ATP6V1B2, STMN2, GABRG2, NDUFB5, GAD1, PGK1, LETMD1, FIBP*	*GOT1, PGK1, AEBP1, NRSN1, FIBP*	*PGK1, AEBP1, NRSN1, SASH1*	*AEBP1, STMN2, SVOP*
	Human osteosarcoma cells (long-term BPA treatment)	3,686	13	0.082	*FIBP, SCG2, TBC1D9, SASH1, GAD2, FOXO1, NME1, NAP1L5, GABRA1, KIFAP3, GAD1, NDRG4, GABRG2*	*GABRA1, FIBP, SCG2*	*FOXO1, SASH1, KIFAP3*	*FOXO1, NME1, SCG2*
GSE86923	Mouse uterine tissues	2,945	13	**0.025**	*NECAP1, ZFP36L1, SNAP25, ATP6V1B2, BCL6, BEX4, NUPR1, MRPL15, SV2B, COPS4, AEBP1, YAP1, SGIP1*	*AEBP1*	*SNAP25, AEBP1*	*BCL6, SNAP25, AEBP1, NUPR1*
GSE102849	Mouse adult hippocampus	310	2	0.548	*SCG2, ATP6V1G2*	*SCG2*	*—*	*SCG2*
GSE103033	Monkey trophoblast stem cells	1,080	6	0.750	*NDUFB5, SNX10, COPS4, MDH1, CD44, ZFP36L1*	*SNX10*	*SNX10*	*SNX10*
GSE121603	Mouse hypothalamus	994	4	0.532	*BCL6, SYTL4, FSTL1, SLC14A1*	*FSTL1, SLC14A1*	*—*	*BCL6, FSTL1*

Hypergeometric distribution analyses were used to analyze associations between significantly differentially expressed genes (DEGs) from other transcriptome profiling studies and AD candidate genes. Statistically significant associations were determined by hypergeometric distribution analysis. P-value < 0.05 is considered as significant.

Furthermore, to determine whether the BPA-responsive genes identified by our study were also dysregulated in the independent studies, the lists of BPA-responsive genes in the hippocampi of rats prenatally exposed to BPA were overlapped with the BPA-responsive genes from the twelve datasets obtained from eight BPA transcriptome studies by other groups. We found that when the list of DEGs in the hippocampus of offspring rats prenatally exposed to BPA from both sexes of rats was overlapped with the lists of DEGs from the previously published transcriptome studies, a total of 1,000 DEGs identified by our study were also found to be dysregulated in at least two other DEG datasets (Supplementary Table S7). Moreover, when each sex was analyzed separately, a total of 416 genes and 744 genes in the hippocampal tissues of BPA-treated male and female pups, respectively, were found to be dysregulated in at least two other DEG datasets (Supplementary Table S8-S9). The lists of overlapping genes between BPA-responsive DEGs in the hippocampus identified by our study and DEGs from the transcriptome studies by other groups were analyzed using IPA software. Similar to our original lists of DEGs, IPA revealed that these overlapping DEGs were significantly associated with several brain disorders, including “neurological disease”, “nervous system development and function”, and “inflammatory response”. The top canonical pathways significantly associated with the overlapping DEGs included “synaptogenesis signaling pathway” and “NF-κB activation”. Moreover, the upstream regulators significantly associated with DEGs in the hippocampus included “TNF”, “TGFB1”, “MAPT”, “APP”, all of which have been associated with AD (P-value < 0.05; Supplementary Table S10).

IPA also revealed specific neurological diseases and multiple processes involved in nervous system development and function that were associated with the overlapping DEGs. We found that the overlapping DEGs were significantly associated with “dementia”, “Alzheimer’s disease”, “cognitive impairment”, “movement disorders”, and” tauopathy”. Our results revealed significant associations between the overlapping DEGs and several nervous system developmental pathways and functions, including “development of neurons”, “neuritogenesis”, “neurotransmission”, and “memory” (P-value < 0.05; Supplementary Table S11). Interestingly, we found that the hub gene in the interactome network generated using the overlapping DEGs was NF-κB (Supplementary Fig. S2). Taken together, the results of these bioinformatic analyses suggest that BPA exposure may cause dysregulation of genes associated with AD-related biological functions in the brain as well as other tissues.

## Discussion

Exposure to BPA during pregnancy has been associated with the risk of several fetal health problems, especially brain and nervous system disorders, and reports have suggested that it not only induces neurotoxicity at early developmental stages but also impacts neuronal morphology during adulthood^19,26,36^. There is accumulating evidence that maternal BPA exposure exerts transgenerational effects that can be observed not only in F1 but also in F2 and F3 offspring^37^. In this study, we investigated the effects of prenatal BPA exposure on transcriptome profiles and AD-related molecular mechanisms in offspring rat brains. We obtained transcriptome profiling data from an RNA-seq analysis of hippocampal tissues isolated from rat offspring prenatally exposed to BPA at the dose of 5,000 μg/kg·maternal BW, which is equal to the No-Observed-Adverse-Effect Level (NOAEL) in humans determined by the FDA and ESFA. The associations between DEGs from the RNA-seq analysis and the top 109 dysregulated genes in the AlzBase database were carried out and revealed that the significantly differentially expressed BPA-responsive genes significantly overlapped with the AD-related genes in the AlzBase database. BPA-responsive DEGs in the hippocampus were significantly associated with several brain disorders, including AD, and biological functions associated with AD, including “neuritogenesis”, “synaptic transmission”, and “memory”. Moreover, BPA-responsive DEGs in the hippocampus were significantly associated with the upstream regulators, particularly Microtubule Associated Protein Tau (MAPT) and Amyloid Beta Precursor Protein (APP), which have been highly implicated in AD neuropathology. Interestingly, we found that the hub protein in the interactome networks is NF-κB, which is a key protein that plays important roles in regulating inflammation, oxidative stress and apoptosis.

Neuroinflammatory cytokines are known to reduce the efflux transport of Aβ, thus leading to elevated Aβ concentrations in the brain and increased susceptibility to AD^34^. NF-κB is currently considered an important agent related to the chronic neuroinflammatory state that persists in the AD brain^38,39^. An increased proportion of neurons with nuclear p65, one of the five components that form the NF-κB transcription factor family and a parameter for NF-κB activation, has been observed in the hippocampus and cortex around Aβ plaques in postmortem AD tissues^40^. We found that the NF-κB protein level was significantly increased in the hippocampi of rats prenatally exposed to BPA. This finding suggests that maternal BPA exposure may induce neuroinflammation and changes in transcriptome profiles in the offspring brain through NF-κB, which should be further investigated. In addition to NF-κB, earlier reports have shown that maternal BPA exposure induced elevation of TNF-α in the dorsal telencephalon, hypothalamus, and prefrontal cortex^41,42^. Here, we found that *Tnf* was also increased in the hippocampus of rats prenatally exposed to BPA. Studies have reported that TNF-α, which can be synthesized in the central nervous system by microglia, neurons, and astrocytes^43–45^, is chronically elevated in the brain, CSF, and blood of AD patients^46–48^. Together, our results suggested that prenatal BPA exposure during pregnancy may induce the elevation of inflammatory cytokines, which may cause chronic neuroinflammation in the hippocampus and other brain regions and, in turn, increase AD susceptibility in the offspring.

BPA can circulate throughout the body and pass through the blood-brain barrier, whereupon it may exert detrimental impacts on brain cells^49^. BPA can also transfer across the placenta to the fetus and is further delivered to offspring through lactation^27,28^. Experimental data indicate that AD-associated pathological proteins were substantially elevated in human and rat neuronal cell models in response to BPA exposure^24^. One of the main pathological hallmarks of AD is the accumulation of neuronal Aβ plaques in the brain, which arises from improper APP cleavage. The resulting Aβ monomers then aggregate, forming oligomeric Aβ and ultimately Aβ fibrils and plaques. This proteolytic processing of APP is mediated by the enzyme beta-secretase 1 (BACE1), which is also known as a beta-site amyloid precursor protein cleaving enzyme. The BACE protein and activity are increased in regions of the brain that develop amyloid plaques in AD^50^. It has been shown that the activation of NF-κB signaling facilitates *BACE1* gene expression and APP processing^51^. Interestingly, we also found that *Bace1* expression was significantly increased in the hippocampi of rat pups prenatally exposed to BPA. Consistent with elevated NF-κB protein level, the overexpression of *Bace1* seems to be specific to male offspring, suggesting that the elevation of NF-κB protein in response to maternal BPA exposure may lead to *Bace1* overexpression in a sex-dependent mannerFang *et al*. (2016). has reported that APP expression and phosphorylated tau were increased in the prefrontal cortex of male offspring mice prenatally exposed to BPA^52^. Therefore, we subsequently investigated the Aβ and APP protein levels in hippocampal tissues and found that while the APP level was not altered, the level of Aβ tended to increase in the male hippocampus, although the difference was not statistically significant. The inconsistency between our findings and the previous report might have been partly due to the variability of different brain regions and limited sample size. Taken together, these results suggest that prenatal BPA exposure during pregnancy can influence the β-secretase enzyme in the amyloidogenic pathways of APP processing in the offspring hippocampus through NF-κB in a sex-dependent manner.

Our data mining analysis of other transcriptome profiling data revealed that the lists of DEGs in response to BPA exposure identified by other groups of researchers also exhibited an overlap with AD candidate genes with a significant association in certain tissues, particularly in placental tissues and fetal mammary glands, suggesting that the effects of maternal BPA exposure on AD-related genes are systemic. Moreover, the BPA-responsive genes identified by our study were also found to be dysregulated in other transcriptome profiling studies. Overlapping DEGs were associated with several biological functions and upstream regulators associated with AD. Moreover, we found that NF-κB is the hub gene in the interactome network generated using the overlapping DEGs. The association of our DEGs and other transcriptome profiling data using adult hippocampus tissues showed little overlap, which was possibly due to the differences in treatment, age, sex, and data analysis. The effects of maternal BPA exposure on AD-related genes, neurological functions, and pathological conditions in adult and older offspring should be further investigated. Maternal BPA exposure has been reported to cause the dysregulation of genes associated with autism^32^, and the findings from this study revealed a possible relationship between AD and autism, inasmuch as susceptibility was linked to the same environmental factor. This suggested relationship deserves further investigation.

In summary, the findings from this study indicate that prenatal BPA exposure impacts transcriptome-interactome profiles of genes in the offspring hippocampus. The differentially expressed genes are significantly associated with AD candidate genes, neuroinflammation, and other regulatory networks known to be impacted in the pathobiology of AD. Moreover, maternal BPA exposure also induced elevation of NF-κB protein and its AD-related target *Bace1*, suggesting that BPA may trigger neuroinflammation in the hippocampus and increase the susceptibility of AD through NF-κB. In addition to the hippocampus, the data mining analysis showed that maternal BPA exposure also disrupted AD-related genes in other tissue types, particularly placental tissues and fetal mammary glands. The underlying mechanisms through which maternal BPA exposure exerts transgenerational, sex-specific effects on neuroinflammation and the brain in the context of AD should be further investigated.

## Methods

### Animal experiments

Animal experiments were approved by the Chulalongkorn University Animal Care and Use Committee (Animal Use Protocol No. 1673007 and No. 1773011), Chulalongkorn University. All protocols were performed according to relevant guidelines and regulations. Eight-week-old female and male Wistar rats were housed at the Chulalongkorn University Laboratory Animal Center (CULAC) under standard temperature (21 ± 1 °C) and humidity (30–70%) conditions in a 12-h light/dark cycle with *ad libitum* access to food and water. Animals were treated as described by Thongkorn *et al*.^32^. Briefly, female rats (gestational day 1 (GD1); n = 8) were divided into 2 groups of 4 each (control group and BPA treatment group). For BPA treatment, BPA (Sigma-Aldrich, USA) was dissolved in absolute ethanol (Merck Millipore, USA) to a final concentration of 250 mg/ml to make a stock BPA solution. Then, the stock solution was further diluted with corn oil to a final concentration of 5,000 μg/kg·maternal BW of BPA to treat each rat. The vehicle control treatment was prepared by mixing absolute ethanol with corn oil in amounts equivalent to those used for preparing the BPA treatment. After mating, each rat was intragastrically administered either BPA or the vehicle control from GD1 until parturition. Neonatal pups were euthanized, and the hippocampi of rat pups prenatally exposed to BPA (male n = 6; female n = 6) or vehicle control (male n = 6; female n = 6) were isolated as previously described^32^. Briefly, neonatal pups were euthanized by decapitation on ice following intraperitoneal injection of 100 mg/kg·BW sodium pentobarbital. The brain was quickly removed from the head and placed in a prechilled tube containing ice-cold, freshly prepared 1X HBSS (Invitrogen, USA) containing 30 mM glucose (Sigma-Aldrich, USA), 2 mM HEPES (GE Healthcare Bio-Sciences, USA), and 26 mM NaHCO_3_ (Sigma-Aldrich, USA). The brain was then dissected, and the hippocampus was isolated under a Nikon SMZ18 Stereo Microscope (Nikon, Japan). Hippocampal tissues were immediately placed in a tube with RNAlater (Ambion, USA) and stored at −80 °C for subsequent analysis. The characteristics of neonatal rat pups are shown in Supplementary Table S12.

### RNA and protein isolation

Total RNA from the hippocampus was isolated and purified using the mirVana miRNA Isolation Kit (Ambion, USA) as previously described by Thongkorn *et al*.^32^. RNA integrity was assessed using an Agilent Bioanalyzer (BGI, Hong Kong). Proteins were extracted from hippocampal tissues using GENEzol reagent (Geneaid Biotech, Ltd., Taiwan) as previously described by Pichitpunpong *et al*. (2019) with slight modifications^53^. Briefly, hippocampal tissues stored in RNAlater were centrifuged, and RNAlater was removed. GENEzol reagent was added to the tissue pellet, and the pellet was homogenized. A total of 100 μL of chloroform was added, followed by centrifugation. The aqueous phase was then removed, and 100% ethanol was added to the interphase and the organic phase. After centrifugation to pellet the DNA, the supernatant was transferred to a new tube for protein isolation. To precipitate proteins, isopropanol was added to the phenol-ethanol supernatant and then centrifuged for 10 min at 12,000 g at 4 °C. The protein pellet was washed 3 times in a wash solution (0.3 M guanidine hydrochloride in 95% ethanol) and then washed once in 100% ethanol. The protein pellet was subsequently air-dried and resuspended in lysis buffer (1% SDS). Protein concentrations were measured using the Bradford protein assay (Bio-Rad, USA).

### Quantitative RT-PCR

First-strand cDNA was generated using the RevertAid First-Strand cDNA Synthesis Kit (Thermo Scientific, USA) following the manufacturer’s protocol. The cDNA synthesis reaction was performed by incubating the reaction at 42 °C for 60 min, followed by 94 °C for 5 min. For the amplification reaction, a quantitative polymerase chain reaction (qPCR) was performed using the Bio-Rad CFX Connect Real-Time System (Bio-Rad, USA). The SYBR green system (Bioneer, Korea) was used to analyze mRNA expression using each gene-specific primer pair. The specific primers in the qPCR analyses were designed using the UCSC In-Silico PCR Genome Browser (https://genome.ucsc.edu/), Ensembl (https://asia.ensembl.org/index.html), and Primer3 software (http://bioinfo.ut.ee/primer3). The primers used in the real-time RT-PCR experiments were as follows: *Bace1* forward primer: 5′-TCACAGTCATCCACAGGCAC-3′, *Bace1* reverse primer: 5′-AGTCTTCCATGTCTGCCGTG-3′, *Tnf* forward primer: 5′-GGCTTTCGGAACTCACTGGA-3′, *Tnf* reverse primer: 5′-GGGAACAGTCTGGGAAGCTC-3′, *Il-1b* forward primer: 5′-GCACAGTTCCCCAACTGGTA-3′, *Il-1b* reverse primer: 5′-GGAGACTGCCCATTCTCGAC-3′, *Il-6* forward primer: 5′-GCTCTGGTCTTCTGGAGTTCC-3′, *Il-6* reverse primer: 5′-AGAGCATTGGAAGTTGGGGT-3′, *Rn18s* forward primer: 5′-CTGGATACCGCAGCTAGGAA-3′, and *Rn18s* reverse primer: 5′-GAATTTCACCTCTAGCGGCG-3′. 18 S ribosomal RNA (*Rn18s*) was used as the housekeeping gene for all genes of interest. Expression was quantified as the fold-change using the ΔΔCt method (2^−ΔΔCt^).

### Western blot analysis

Protein concentrations were measured using a protein assay (Bio-Rad, USA). Equal amounts of protein (15 µg) were separated by 10% SDS-PAGE and transferred to PVDF blotting membranes. The membranes were blocked with TBS-T containing 5% nonfat dry milk for 1 h before incubation with primary antibodies against APP (1:5000, Cell Signaling Technology, Inc., USA), β-Amyloid 1–42 Specific (1:7500, Cell Signaling Technology, Inc., USA), or transcription factor nuclear factor-κ-light-chain-enhancer of activated B cells (NFκB, 1:7500, Cell Signaling Technology, Inc., USA) overnight at 4 °C. Then, membranes were incubated with secondary antibodies (donkey anti-rabbit or anti-mouse IgG HRP (1:10000 and 1:15000, Abcam, UK). The blots were incubated in Amersham ECL Select Western Blotting Detection Reagent (GE healthcare, UK), and the membranes were then visualized using high-performance chemiluminescence film (GE healthcare, UK). The protein band density was determined using ImageJ software (National Institutes of Health) for densitometry, and β-actin (1:10000; Santa Cruz Biotechnology, USA) was used as the housekeeping protein.

### Transcriptome data collection

The transcriptome profiling data of total RNA isolated from the hippocampal tissues of offspring rats from six independent litters prenatally exposed to BPA or the vehicle control were obtained from the NCBI GEO database (GEO DataSets: http://www.ncbi.nlm.nih.gov/gds) with the accession number: GSE140298. In addition, we also obtained 12 other transcriptome profiling datasets from eight studies of human or animal models exposed to BPA or the vehicle control using microarray or RNA sequencing platforms from GEO DataSets. The details of each study are provided in Supplementary Table S6. Only transcriptome profiling data of cells or tissues exposed to BPA or BPA-vehicle controls were used prior to subsequent BPA-responsive gene identification.

### Identification of differentially expressed genes in response to BPA

In brief, transcriptome profiling was performed by BGI Genomics Co., Ltd. using the Illumina HiSeq. 4000 next-generation sequencing platform with 4 G reads (Illumina, Inc.) according to the manufacturer’s protocol. Total RNA was treated with DNase I, and the oligo(dT) treatment was used for mRNA isolation. Next, the RNA was mixed with fragmentation buffer to fragment the mRNA. Then, cDNA was synthesized using the mRNA fragments as templates. Subsequently, sequencing reads were filtered and subjected to quality control. Clean reads in a FASTQ file were mapped to the rat reference genome (RefSeq ID: 1174938) using Bowtie 2^54^, and gene expression levels were then calculated using RSEM^55^. We then compared the transcriptome profiles between the BPA and the control groups with a Poisson distribution. Comparisons were performed with all male and female pups with the same treatment condition combined into one group and separately for each sex. P-values were calculated using a Poisson distribution method. DEGs with a P-value < 0.05 and FDR < 0.05 were considered statistically significant.

To identify BPA-responsive genes from previously published studies, the transcriptome profiling data from the microarray platform were re-analyzed separately by the Multiple Experiment Viewer (MeV) program as previously described^56–58^. Briefly, the transcriptome data were filtered using a 70% cutoff function, which removes transcripts for which intensity values are missing in more than 30% of the samples. All studies compared the BPA-treated group versus the untreated/vehicle control in the analyses. The available transcripts were then used for identifying DEGs by the t-test permutation (α = 0.05) with standard Bonferroni correction. For RNA-seq-based transcriptome studies, we obtained all DEGs (P-value < 0.05) that were provided in the original article. The lists of DEGs from each study were used to determine the association with Alzheimer’s disease candidate genes by hypergeometric distribution analysis.

### Hypergeometric distribution analysis

The list of AD candidate genes was obtained from a summary of the top 109 genes from AlzBase (details available at the AlzBase Web site)^59^. We overlapped the lists of significantly differentially expressed BPA-responsive genes in the offspring hippocampus and AD-related genes (the top 109 DEGs with a frequency greater than 15 in the AlzBase database, http://alz.big.ac.cn/alzBase). Then, the associations between BPA-responsive DEGs and AD candidate genes were assessed via a hypergeometric distribution analysis (https://keisan.casio.com/exec/system/1180573201). These processes were repeated with the lists of DEGs from each previously published study. A hypergeometric P-value of less than 0.05 was considered significant.

### Prediction of biological functions and interactome analysis

Biological functions, disorders, canonical pathways, and interactome networks associated with DEGs were predicted using IPA software (Qiagen, Inc., USA). The list of DEGs were compared against genes in the Ingenuity Knowledge Base which contains all experimentally validated genes that are associated with each specific function, disorder, or canonical pathway. Fisher’s exact test was then performed to calculate P-values for enrichment with respect to a particular pathway, function, or disorder, and a P-value < 0.05 was considered statistically significant.

### Statistical analysis

The results are presented as the mean ± SEM. The differences between two groups were analyzed by a two-tailed Student’s t-test using SPSS version 22.0. The differences were considered statistically significant at a P-value < 0.05.

## Supplementary information

Supplementary Information.

## Data Availability

The transcriptome profiling data used in this study have been published in the NCBI GEO DataSets database (GSE140298, GSE44387, GSE63852, GSE58642, GSE50527, GSE86923, GSE102849, GSE103033, and GSE121603).
